# Identification of Somatic and Germline Mutations Influencing Treatment Outcomes and Disease Susceptibility in Tunisian Triple-Negative Breast Cancer Using Next-Generation Sequencing

**DOI:** 10.3389/bjbs.2026.15988

**Published:** 2026-04-22

**Authors:** Asma Mehri, Ahmed Baligh Laaribi, Ichraf Jbir, Emna Chelbi, Beya Chelly, Abir Chaabane, Salwa Nechi, Hadda-Imen Ouzari

**Affiliations:** 1 Laboratory of Microorganisms and Active Biomolecules (LR03ES03), Faculty of Sciences of Tunis, University of Tunis El Manar, Tunis, Tunisia; 2 Commun Sequencing Unite, Department of Biology, Faculty of Sciences of Tunis, University of Tunis El Manar, Tunis, Tunisia; 3 Faculty of Medicine of Tunis, University of Tunis El Manar, Tunis, Tunisia; 4 Department of General Surgery, University Hospital of Mohamed Taher Maamouri, Nabeul, Tunisia; 5 Department of Pathology, University Hospital of Mohamed Taher Maamouri, Nabeul, Tunisia

**Keywords:** germline variants, next-generation sequencing (NGS), precision medicine, somatic mutations, TP53

## Abstract

Triple-negative breast cancer (TNBC) is an aggressive breast cancer subtype characterized by marked molecular heterogeneity and limited targeted therapeutic options. Its incidence is rising in many low- and middle-income countries, where genetic profiling of affected patients remains largely unexplored despite evident clinical disparities. This study aimed to characterize, for the first time in a Tunisian cohort, the spectrum of germline and somatic mutations in TNBC patients and to assess their potential impact on therapeutic response. Targeted next-generation sequencing (NGS) of hotspot regions across 50 cancer-related genes was performed in twelve patients using the AmpliSeq for Illumina Cancer Hotspot Panel v2, applied to both tumor tissues and matched adjacent non-tumoral tissues. Bioinformatics analysis revealed recurrent germline variants present in all samples, notably in TP53 (rs1042522), CSF1R (rs2066933), FGFR3 (rs7688609), RET (rs1800861), KDR (rs7692791), and PDGFRA (rs1873778). In tumor tissues, 32 deleterious somatic variants were detected across 20 oncogenes, with TP53 emerging as the most frequently mutated gene (58%). Distinct mutational patterns were observed in relation to treatment response. Notably, the co-occurrence of AKT1 (rs121434592) and TP53 (rs876660754) was observed in a patient with treatment resistance, whereas an in-frame deletion in NOTCH1 (p.Val1578del) was uniquely detected in patients who subsequently experienced disease recurrence. These findings provide the first comprehensive characterization of germline and somatic alterations in Tunisian TNBC patients, representing a North African cohort. They reveal the heterogeneity of mutation patterns linked to treatment response, and emphasize the importance of genomic profiling into clinical practice and guide personalized therapeutic strategies.

## Introduction

Breast cancer (BC) remains the most commonly diagnosed malignancy and the leading cause of cancer-related mortality among women worldwide, accounting for more than 2.3 million new cases and over 670,000 deaths annually [1, 2]. Although incidence rates are traditionally higher in high-income countries, a rapid and alarming increase has been observed in low- and middle-income regions, where more than 60% of global breast cancer deaths now occur due to limited access to early detection and effective treatment infrastructures [2]. Among the molecular subtypes, triple-negative breast cancer (TNBC), defined by the absence of estrogen receptor, progesterone receptor, and HER2 expression, represents approximately 10%–20% of all breast cancers and is disproportionately more common in younger women, African populations, and low-resource settings [3, 4]. TNBC is characterized by its marked biological heterogeneity, rapid progression, high metastatic potential, and poor clinical outcome compared to other breast cancer subtypes [5]. Its intrinsic aggressiveness, combined with the lack of targeted hormonal or HER2-directed therapies, positions TNBC as a major global health challenge, particularly in countries where delayed diagnosis and constrained therapeutic options further worsen prognosis.

The advent of next-generation sequencing (NGS) technologies has revolutionized cancer genomics by enabling high-throughput, cost-efficient, and highly sensitive profiling of tumor genomes. This advancement has dramatically accelerated the discovery of pathogenic variants across diverse cancer types, including TNBC, where genomic instability and mutational heterogeneity are defining features [6, 7]. Through targeted panels, whole-exome sequencing, and whole-genome approaches, NGS has uncovered recurrent somatic driver mutations, particularly in TP53, PI3K/AKT, and DNA-repair pathways, that shape tumor behavior, therapeutic response, and clinical outcome [8]. Beyond somatic alterations, NGS has also highlighted the critical role of germline variants in modulating cancer susceptibility and influencing tumor biology, with BRCA1/2 and other DNA-repair gene mutations disproportionately represented in TNBC [9, 10].

However, despite these scientific advances, a major global imbalance persists: more than 80% of genomic studies in breast cancer have been conducted in European or North American populations, whereas African and North African populations remain profoundly underrepresented [11]. Limited access to NGS platforms, financial constraints, and scarce research infrastructure in low- and middle-income countries continue to hinder the generation of large-scale genomic datasets from African women [12]. This lack of representation is particularly concerning for TNBC, which is known to be more prevalent and more aggressive among women of African ancestry. Therefore, defining the mutational landscape in African and North African populations is essential to decipher population-specific drivers, identify convergent oncogenic pathways across ethnic groups, and ultimately improve precision oncology approaches tailored to genetically diverse populations [13].

Despite this gap, no comprehensive genomic study has yet been conducted in Tunisian women with TNBC, and available data from North Africa remain extremely scarce and fragmented. This lack of molecular information poses a significant challenge, as population-specific genetic backgrounds may influence both cancer susceptibility and the spectrum of actionable or prognostic mutations. In this context, the use of a clinically oriented 50-oncogene panel represents a pragmatic and powerful approach for simultaneously interrogating key cancer-related pathways while remaining accessible in resource-limited settings.

A deeper understanding of the molecular and genetic architecture of TNBC in this population is essential for advancing precision oncology in underrepresented regions. Therefore, our study aims to characterize, for the first time, the combined germline and somatic mutational landscape of TNBC in Tunisian patients using a targeted NGS approach, with the objective of generating population-specific genomic insights that may inform risk assessment, early detection strategies, and tailored therapeutic decision-making.

## Materials and Methods

### Patients and Samples Collection

This retrospective study was conducted as part of a broader research program aimed at characterizing the genetic landscape of breast cancer in Tunisian patients. Participants were randomly selected from the Department of Surgical Oncology at Mohamed Taher Maamouri University Hospital in Nabeul, Tunisia. Patients who underwent surgical resection between 2023 and 2024 were included if they had confirmed diagnosis of non-metastatic, histologically malignant epithelial breast tumor based on core needle biopsy. Written informed consent was obtained from all participants.

Among approximately 120 patients initially screened, twelve patients with confirmed TNBC were selected based on histopathological diagnosis. Immunohistochemistry analysis was performed using the fully automated Bond Max platform (Leica, Biosystems, Buccinasco, Milan, Italy). TNBC status was defined according to American Society of Clinical Oncology/College of American Pathologists (ASCO/CAP) guidelines as estrogen receptor (ER) expression <1% and progesterone receptor (PR) expression <1%. HER2 status was assessed as 0, 1+, or 2+ with negative chromogenic *in situ* hybridization (CISH) confirmation for equivocal cases. Clinical data were retrieved from patient medical records.

The study included eight surgically resected TNBC tissue samples collected after completion of neoadjuvant chemotherapy, along with matched adjacent non-tumor tissues. Four additional samples were obtained as formalin-fixed, paraffin-embedded (FFPE) specimens stored for less than 1 year. All Tissue sampling was performed under the supervision of a certified pathologist to ensure accurate tumor content assessment and proper handling. Immediately after surgical resection, all fresh tissue samples were immersed in RNAprotect® Tissue Reagent (QIAGEN, Hilden, Germany) and stored at −20 °C until nucleic acid extraction.

The study was conducted in accordance with the ethical principles of the Declaration of Helsinki and was approved by the Ethics Committee of Mohamed Taher Maamouri University Hospital in Nabeul (BC-01/2023).

### Genomic DNA Extraction and Quality Control

Genomic DNA was extracted from fresh-frozen and FFPE tumor tissues using QIAGEN purification systems (QIAGEN, Hilden, Germany). DNA from fresh-frozen tissues was isolated using the QIAamp® DNA Mini Kit, while DNA extraction from FFPE samples was performed using the QIAamp® DNA FFPE Tissue Kit. All procedures were carried out strictly according to the manufacturer’s protocols to ensure high yield and purity.

DNA concentration was measured using the DeNovix QFX Fluorometer (DeNovix Inc., Wilmington, DE, USA) in combination with the Qubit™ dsDNA High-Sensitivity Assay Kit (Thermo Fisher Scientific, Waltham, MA, USA). DNA quality and integrity were evaluated using the Agilent 2100 Bioanalyzer (Agilent Technologies, Santa Clara, CA, USA), enabling assessment of fragment size distribution and sample suitability for downstream NGS applications.

### Targeted Sequencing

Targeted NGS was carried out with the AmpliSeq for Illumina Cancer Hotspot Panel v2 (Illumina, San Diego, CA, USA), designed to amplify 207 specific amplicons encompassing roughly 2800 known mutations in the hotspot regions of 50 cancer-related genes. According to the manufacturer’s instructions, libraries were constructed from 100 ng of genomic DNA per sample using the AmpliSeq™ Library PLUS for Illumina® kit. Briefly, the procedure involved multiplex PCR amplification for target enrichment, followed by partial digestion of primer sequences and adapter ligation using the AmpliSeq™ CD Indexes Set A for Illumina® to assign unique barcodes to each library.

Libraries were purified using Agencourt® AMPure® XP beads (Beckman Coulter Inc., Brea, CA, USA). DNA concentration was quantified with the DeNovix QFX Fluorometer and fragment size profiles were further assessed using the Agilent 2100 Bioanalyzer (Agilent Technologies, Santa Clara, CA, USA). Libraries meeting quality requirements were normalized and pooled in equimolar ratios.

Prior to sequencing, the library pool was diluted to a final loading concentration of 8 pM and supplemented with a 5% PhiX Control v3 (Illumina) to enhance diversity and serve as an internal control. The pool was sequenced on an Illumina MiSeq platform equipped with a MiSeq Reagent Kit v2 (300-cycle) for 2 × 150 bp paired-end sequencing. The sequencing run was monitored using the Illumina Local Run Manager, with key metrics such as the percentage of bases ≥ Q30, mean coverage depth, and uniformity of coverage assessed against the manufacturer’s specifications. Only data passing these quality thresholds were subjected to subsequent bioinformatics analyses.

### Bioinformatics Analysis

Raw sequencing data (FASTQ files) were processed using the DRAGEN Amplicon Pipeline on the Illumina BaseSpace Sequence Hub. Reads were aligned to the human reference genome (GRCh37/hg19). Somatic variant calling was performed using the DRAGEN Somatic Pipeline, which analyzes matched tumor-normal sample pairs to identify somatic mutations by filtering out germline variants present in the normal sample.

Functional annotation of the filtered variants was performed using SnpEff implemented on the Galaxy platform. The SnpEff workflow was configured with the hg19/GRCh37 reference database, enabling systematic classification of each variant according to its predicted molecular consequence. All shortlisted somatic variants were manually verified using the Integrative Genomics Viewer (IGV, version 2.16.2 07/2023) to inspect read alignment and rule out technical artefacts.

The final curated list of high-confidence variants was analyzed and visualized in RStudio (Version 2024.12.0+467). The maftools (version 2.12.0) and ComplexHeatmap packages (version 2.12.1) were used to generate comprehensive oncoplots, respectively, while additional custom plots were created using ggplot2 (version 3.5.1).

### Protein-Protein Interaction Network Construction and Clustering Analysis

We constructed a protein-protein interaction (PPI) network to elucidate potential functional interactions among the mutated genes identified in our TNBC cohort. The network was generated using the STRING database (version 12.0) by inputting the respective gene symbols and executing the analysis with all default settings. Subsequently, the network was partitioned into clusters using the Markov Cluster Algorithm (MCL), applying the default inflation parameter of 3 to define protein complexes and functional modules. Finally, each cluster was annotated for biological pathway involvement via the built-in STRING enrichment analysis tool, with a focus on Gene Ontology (GO) pathways.

### Statistical Analysis

Descriptive statistical analyses were performed to summarize the demographic and clinical characteristics of the study cohort. Continuous variables were reported as mean ± standard deviation (SD). Categorical variables were expressed as frequencies and percentages. All statistical analyses were conducted using GraphPad Prism v10.0 (GraphPad Software, San Diego, CA, USA).

## Results

### Demographic and Clinical Characteristics of Study Population

A total of twelve patients diagnosed with TNBC were included in this study. The demographic and clinicopathological characteristics are summarized in Table 1. The mean age at diagnosis was 58.3 ± 2.7 years, with ages ranging from 47 to 76 years. Tumor sizes varied between 4 mm and 40 mm, and the majority of tumors presented a high histological grade, with an SBR grade of 3 observed in most cases. Proliferative activity was markedly elevated, as reflected by a Ki-67 index ranging from 15% to 90%. Regarding treatment response, two patients achieved a pathological complete response (pCR = 100%), while four patients showed a good therapeutic response (>50%). The remaining patients exhibited partial response (<50%) or complete resistance (0%). Additionally, two patients developed tumor recurrence, one occurring 1 year after treatment and the other 5 years later. The cohort included eight fresh-frozen tissue samples and four FFPE samples, providing a representative set of routinely available clinical materials for molecular analysis.

**TABLE 1:** Demographic and clinicopathological characteristics of TNBC patients.

ID	Tumor size (mm)	SBR grade	Ki67 index (%)	pCR (%)	Type of sample	Age
1	25	2	60	100	Fresh tissue	63
2	12	3	60	100	Fresh tissue	55
3	22	2	45	>50	Fresh tissue	48
4	5	3	80	>50	Fresh tissue	56
5	17	3	90	>50	FFPE	52
6	4	3	15	>50	FFPE	76
7	15	2	50	>50	FFPE	61
8	32	3	60	<50	Fresh tissue	56
9	11	3	25	<50	FFPE	51
10	32	3	60	0	Fresh tissue	47
11	40	3	65	R	Fresh tissue	75
12	25	3	70	R	Fresh tissue	60

SBR, Scarff-Bloom-Richardson; pCR, pathologic complete response.

### Germline Variants Profile Identified in Our TNBC Cohort

**FIGURE 1:**
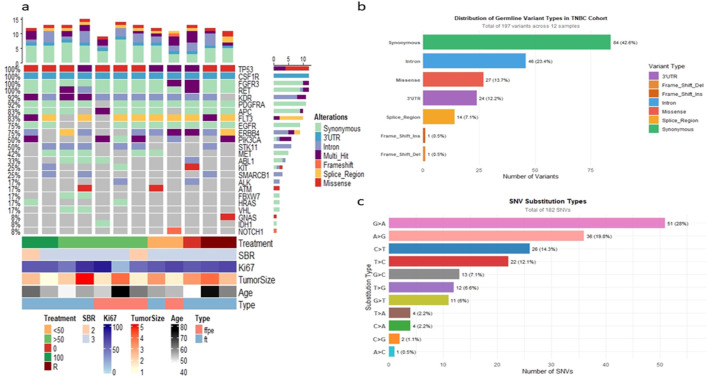
Germline variant landscape in the TNBC cohort. **(a)** Oncoplot illustrating oncogenic germline variants identified across TNBC patients. Each column represents an individual patient, and each row corresponds to a cancer-associated gene. **(b)** Bar chart summarizes the distribution of germline variant types, highlighting the predominance of synonymous and intronic variants. **(c)** Bar chart presents the spectrum of SNV substitution types observed in the cohort.

Germline variant analysis across the twelve TNBC patients revealed a diverse set of inherited alterations, predominantly classified as benign or likely benign according to ClinVar, yet reflecting a broad genomic variability within the cohort (Table 2). The most frequently mutated gene was TP53 p.Pro72Arg (rs1042522) variant, detected in all patients (100%). Other genes with high mutation prevalence included CSF1R (rs2066933), FGFR3 (rs7688609), RET (rs1800861), KDR (rs7692791), and PDGFRA (rs1873778), each altered in over 80% of the cohort. Additional recurrently affected genes included APC (rs41115), FLT3 (rs2491231), EGFR (rs1050171), ERBB4 (rs839541), and PIK3CA (rs3729674), showing mutation frequencies ranging from 50% to 75%. Less frequently mutated genes, such as MET, ABL1, KIT, SMARCB1, ALK, ATM, FBXW7, VHL, HRAS, GNAS, IDH1, and NOTCH1, were identified in 8%–33% of patients (Figure 1a).

**TABLE 2:** Germline mutations detected in TNBC patients.

Gene	Position (hg19)	Variant	Variant type	rs ID	Pathogenicity (ClinVar)	Number of cases (%)	MAF (gnomAD)
IDH1	Chr2: 209113192	NM_005896.4 c.315C>T p.Gly105 =	Synonymous Exon: 4	rs11554137	Benign	1/12 (8)	0.05
ERBB4	Chr2: 212578380	NM_005235.2 c.884-7del	Splice region Intron	rs67894136	Benign	4/12 (33)	0.017
ERBB4	Chr2: 212812097	NM_005235.2 c.421+58A>G	Intron	rs839541	Benign	7/12 (58)	0.26
ALK	Chr2: 29432625	NM_004304.4 c.3836+27G>T	Intron	rs3738868	Benign	2/12 (17)	0.019
VHL	Chr3: 10183852	NM_000551.4 c.321C>A p.Arg107 =	Synonymous Exon: 1	NA	VUS	2/12 (17)	NA
PIK3CA	Chr3: 178917005	NM_006218.3 c.352+40A>G	Intron	rs3729674	Benign	5/12 (42)	0.2
PIK3CA	Chr3: 178927410	NM_006218.3 c.1173A>G p.(Ile391Met)	Missense Exon: 7	rs2230461	Benign	4/12 (33)	0.06
PIK3CA	Chr3: 178916823	NM_006218.3 c.210C>T p.Phe70 =	Synonymous Exon: 2	rs760094170	Likely benign	1/12 (8)	<0.001
FGFR3	Chr4: 1806131	NM_000142.5 c.1150T>C p.Phe384Leu	Missense Exon: 9	rs17881656	Benign	2/12 (17)	0.004
FGFR3	Chr4: 1807894	NM_001163213.1 c.1959G>A p.(Thr653 =)	Synonymous Exon: 14	rs7688609	Likely benign	12/12 (100)	0.998
PDGFRA	Chr4: 55152040	NM_006206.5 c.2472C>T p.(Val824 =)	Synonymous Exon: 18	rs2228230	Benign	4/12 (33)	0.16
PDGFRA	Chr4: 55141055	NM_006206.5 c.1701A>G p.(Pro567 =)	Synonymous Exon: 12	rs1873778	Benign	11/12 (92)	0.992
KIT	Chr4: 55593464	NM_000222.3 c.1621A>C p.(Met541Leu)	Missense Exon: 10	rs3822214	Benign	1/12 (8)	0.095
KIT	Chr4: 55593481	NM_000222.3 c.1638A>G p.Lys546 =	Synonymous Exon: 10	rs55986963	Benign	1/12 (8)	0.027
KIT	Chr4: 55597845	NM_000222.3 c.2142-36A>G	Intron	rs17084713	Benign	1/12(8)	0.004
KDR	Chr4: 55980239	NM_002253.3 c.798+54G>A	Intron	rs7692791	Benign	10/12 (83)	0.541
KDR	Chr4: 55972974	NM_002253.3 c.1416A>T p.Gln472His	Missense Exon: 11	rs1870377	Benign	3/12 (25)	0.227
FBXW7	Chr4: 153247278	NM_001349798.2 c.1524A>G p.(Gln428 =)	Synonymous Exon: 9	rs147462419	VUS	2/12 (17)	<0.001
APC	Chr5: 112175770	NM_000038.6 c.4479G>A p.(Thr1493 =)	Synonymous Exon: 16	rs41115	Benign	9/12 (75)	0.625
APC	Chr5: 112175617	NM_000038.6 c.4326T>A p.Pro1442 =	Synonymous Exon: 15	rs67622085	Benign	1/12 (8)	0.01
CSF1R	Chr5: 149433596	NM_005211.3 c.*35_*36delinsTC	3-prime UTR Exon: 22	rs2066933	Benign	12/12 (100)	0.769
EGFR	Chr7: 55249063	NM_005228.5 c.2361G>A p.(Gln787 =)	Synonymous Exon: 20	rs1050171	Benign	8/12 (67)	0.558
MET	Chr7: 116339672	NM_000245.4 c.534C>T p.(Ser178 =)	Synonymous Exon: 2	rs35775721	Benign	5/12 (42)	0.048
ABL1	Chr9: 133738377	NM_005157.6 c.777C>T p.Gly278	Synonymous Exon: 4	rs754813636	Likely benign	3/12 (25)	<0.001
ABL1	Chr9:133747457	NM_005157.6 c.823-59C>T	Intron	rs35093322	Benign	1/12 (8)	0.002
NOTCH1	Chr9: 139,390,861	NM_017617.5 c.7314_7330del p.Ser2439Alafs*62	Frame Shift	NA	VUS	1/12 (8)	NA
RET	Chr10: 43613843	NM_020975.6 c.2307G>T p.(Leu769 =)	Synonymous Exon: 13	rs1800861	Benign	11/12 (92)	0.75
RET	Chr10: 43617317	NM_020975.6 c.2731-76del	Intron	NA	VUS	2/12 (17)	NA
RET	Chr10: 43609942	NM_020975.6 c.1894G>A p.Glu632Lys	Missense Exon: 11	rs377767407	VUS	1/12 (8)	<0.001
HRAS	Chr11: 534242	NM_005343.3 c.81T>C p.(His27 =)	Synonymous Exon: 2	rs12628	Benign	2/12 (17)	0.33
ATM	Chr11:108138003	NM_000051.4 c.2572T>C p.Phe858Leu	Missense Exon: 17	rs1800056	Benign/Likely benign	2/12 (17)	0.01
FLT3	Chr13: 28602292	NM_004119.2 c.2053+23A>G	Intron	rs75580865	Benign	2/12 (33)	0.062
FLT3	Chr13: 28610183	NM_004119.3 c.1310-3T>C	Splice region Intron	rs2491231	Benign	10/12 (84)	0.74
TP53	Chr17: 7578210	NM_000546.6 c.639A>G p.(Arg213 =)	Synonymous Exon: 6	rs1800372	Benign	4/12 (33)	0.014
TP53	Chr17: 7579472	NM_000546.6 c.215C>G p.(Pro72Arg)	Missense Exon: 4	rs1042522	Benign	12/12 (100)	0.7
STK11	Chr19 : 1220321	NM_000455.45 c.465-51T>C	Intron	rs2075606	Benign	6/12 (50)	0.25
GNAS	Chr20: 57484597	NM_000516.7 c.681G>C p.Gln227His	Missense Exon: 8	rs137854533	Pathogenic	1/12 (8)	<0.001
SMARCB1	Chr22: 24176287	NM_003073.5 c.1119-41G>A	Intron	rs5030613	Benign	3/12 (25)	0.13

Analysis of variant types revealed that the majority of alterations were synonymous (42.6%, 84/197) or intronic (23.4%, 46/197), together accounting for approximately 67% of variants that are unlikely to affect protein structure Potentially consequential variants were less frequent: missense mutations represented 13.7% (27/197) of all germline variants, while putative loss-of-function alterations, including frameshift insertions and deletions, were rare (1.0% combined). Additionally, 7% of variants affected 3′UTR regions, which can influence gene transcription and post-transcriptional regulation. Collectively, around 23% of the germline variants identified may play a pivotal role in TNBC susceptibility (Figure 1b).

This profile highlights a recurrent oncogenic germline variant landscape within our TNBC cohort, underscoring the need for investigation in larger patient populations to better elucidate their contribution to inherited susceptibility in this aggressive breast cancer subtype.

Furthermore, the analysis of single nucleotide variation (SNV) substitution types revealed that the most frequent substitution observed was G>A, which accounted for 28% of all SNVs (n = 51). This was followed closely by the A>G substitution, which represented 19.8% of the total (n = 36), and C>T substitutions, contributing 14.3% (n = 26). Cumulatively, these three transition types (G>A, A>G, and C>T) constituted 62.1% of the total SNV burden. Conversely, transversions (purine to pyrimidine or pyrimidine-to-purine changes) were less frequent. The intermediate substitutions included T>C (12.1%, n = 22), G>C (7.1%, n = 13), and T>G (6%, n = 11). The least common substitution type was A>C, observed only once (0.5%), followed by other low-frequency events such as T>A (2.2%, n = 4) and C>A (2.2%, n = 4). The observed signature, characterized by a high proportion of G>A and A>G transitions, suggests the presence of a specific underlying mutational process shaping the germline variant landscape in the analyzed TNBC samples (Figure 1c).

### Somatic Mutations Profile Identified in Our TNBC Cohort

**FIGURE 2:**
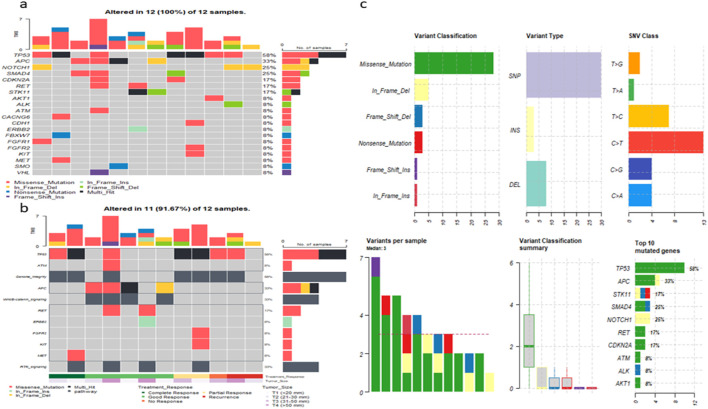
Somatic variant landscape in the TNBC cohort. **(a)** Oncoplot displaying hotspot deleterious somatic mutations that passed stringent filtering criteria and were detected exclusively in TNBC tumor tissues. **(b)** Oncoplot showing the distribution of somatic mutations across key oncogenic pathways in relation to the clinical characteristics of TNBC patients. **(c)** PlotMAF summary illustrating global somatic mutation metrics, including variant classification, variant types, SNV substitution patterns and top 10 mutated genes in our cohort.

Somatic variant analysis in the twelve TNBC patients revealed a heterogeneous mutational landscape affecting multiple cancer-related genes (Table 3). A total of 32 deleterious somatic variants across 20 oncogenes were identified. Analysis of the somatic mutational landscape revealed a median of 3 deleterious mutations per tumor (Figure 2a). The vast majority of variants were single nucleotide polymorphisms (SNPs), with a strong predominance of C>T transitions, followed by T>C and C>G substitutions, consistent with mutational signatures commonly observed in solid tumors. Insertions (INS) and deletions (DEL) collectively accounted for a minor fraction of the total variants. These somatic variants exhibited variable predicted pathogenicity according to ClinVar and AMP classification, reflecting the complex genomic heterogeneity characteristic of TNBC (Figure 2c).

**TABLE 3:** Somatic mutation in TNBC patients.

Gene	Position (hg19)	Variant	Variant type	rs ID	Pathogenicity (ACMG)	AMP classification	Number of cases (%)
ALK	Chr2: 29443610	NM_004304.5 c.3607del p.Asp1203Thrfs*55	Frame_Shift_Del Exon:23	Novel	VUS	Tier3	1/12 (8)
VHL	Chr3: 10183856	NM_000551.4 c.326_327insTT p.His110Serfs*50	Frame_Shift_Ins Exon: 1	Novel	Likely pathogenic	Tier1	1/12 (8)
KIT	Chr4: 55599288	NM_000222.3 c.2414T>A p.Ile805Asn	Missense Exon: 17	Novel	VUS	Tier3	1/12 (8)
FBXW7	Chr4: 153258986	NM_001349798.2 c.829C>T p.Gln277*	Nonsense Exon: 4	Novel	Likely pathogenic	Tier2	1/12 (8)
APC	Chr5: 112175476	NM_000038.6 c.4186_4188del p.Phe1396del	In_Frame_Del Exon: 16	NA	VUS	Tier3	1/12 (8)
APC	Chr5: 112175589	NM_000038.6 c.4298C>T p.Pro1433Leu	Missense Exon: 15	NA	VUS	Tier3	2/12 (17)
APC	Chr5: 112175615	NM_000038.6 c.4324C>G p.Pro1460Ala	Missense Exon: 16	Novel	VUS	Tier3	1/12 (8)
MET	Chr7: 116423407	NM_000245.4 c.3682G>A p.Asp1246Asn	Missense Exon: 19	rs121913671	Likely pathogenic	Tier2	1/12 (8)
SMO	Chr7: 128845086	NM_005631.5 c.580G>T p.Glu194*	Missense Exon: 3	Novel	VUS	Tier3	1/12 (8)
FGFR1	Chr8: 38285931	NM_023110.3 c.381T>G p.Asp127Glu	Missense Exon: 4	rs750795714	VUS	Tier2	1/12 (8)
NOTCH1	Chr9: 139399411	NM_017617.5 c.4732_4734del p.Val1578del	In_Frame_Del Exon: 26	rs761020817	VUS	Tier2	3/12 (25)
CDKN2A	Chr9: 21971066	NM_000077.5 c.292C>G p.His98Asp	Missense Exon: 2	Novel	VUS	Tier3	1/12 (8)
CDKN2A	Chr9: 21971111	NM_000077.5 c.247C>T p.His83Tyr	Missense Exon: 2	rs121913385	Likely pathogenic	Tier1	1/12 (8)
RET	Chr10: 43610011	NM_020975.6 c.1963T>C p.Phe655Leu	Missense Exon: 11	rs756978792	Likely pathogenic	Tier2	1/12 (8)
RET	Chr10: 43615569	NM_020975.6 c.2648C>T p.Ala883Val	Missense Exon: 15	rs1293645997	Likely pathogenic	Tier1	1/12 (8)
FGFR2	Chr10: 123258005	NM_000141.5 c.1672+4A>G	Missense Exon: 12	Novel	VUS	Tier3	1/12 (8)
ATM	Chr11: 108204634	NM_000051.4 c.7949A>C p.Asp2650Ala	Missense Exon: 54	rs1060501635	VUS	Tier3	1/12 (8)
AKT1	Chr14: 105246550	NM_001382430.1 c.49G>A p.Glu17Lys	Missense Exon: 4	rs121434592	Pathogenic	Tier1	1/12 (8)
CDH1	Chr16: 68846039	NM_004360.5 c.1010G>A p.Ser337Asn	Missense Exon: 4	Novel	VUS	Tier3	1/12 (8)
TP53	Chr17: 7578290	NM_000546.6 c.560-6_560-1del	Splice region Del	-	Likely pathogenic	Tier1	1/12 (8)
TP53	Chr17: 7578413	NM_000546.6 c.517G>T p.Val173Leu	Missense Exon: 5	rs876660754	Pathogenic	Tier1	1/12 (8)
TP53	Chr17: 7578461	NM_000546.6 c.469G>T p.Val157Phe	Missense Exon: 5	rs121912654	Pathogenic	Tier1	2/12 (17)
TP53	Chr17: 7578550	NM_000546.6 c.380C>T p.Ser127Phe	Missense Exon: 5	rs730881999	Pathogenic	Tier1	2/12 (17)
TP53	Chr17: 7578190	NM_000546.6 c.659A>G p.Tyr220Cys	Missense Exon: 6	rs121912666	Pathogenic	Tier1	1/12 (8)
TP53	Chr17: 7578272	NM_000546.6 c.577C>T p.His193Tyr	Missense Exon: 6	rs876658468	Pathogenic	Tier1	1/12 (8)
TP53	Chr17: 7577565	NM_000546.6 c.716A>G p.Asn239Ser	Missense Exon: 6	rs1057519999	Pathogenic	Tier1	1/12 (8)
TP53	Chr17: 7577578	NM_000546.6 c.703A>G p.Asn235Asp	Missense Exon: 7	Novel	Likely pathogenic	Tier1	1/12 (8)
TP53	Chr17: 7577121	NM_000546.6 c.817C>T p.Arg273Cys	Missense Exon: 8	rs121913343	Pathogenic	Tier1	1/12 (8)
ERBB2	Chr17: 37881454	NM_004448.4 c.2646delinsAA p.Val884Glyfs*21	In_Frame_Ins	Novel	Likely pathogenic	Tier3	1/12 (8)
SMAD4	Chr18: 48604641	NM_005359.6 c.1463C>T p.Ala488Val	Missense Exon: 12	Novel	VUS	Tier3	2/12 (17)
SMAD4	Chr18: 48593530	NM_005359.6 c.1281del p.His427Glnfs*9	Frame_Shift_Del	Novel	Likely pathogenic	Tier2	1/12 (8)
CACNG6	Chr19: 54496289	NM_145814.2 c.158C>T p.Ala53Val	Missense Exon: 1	Novel	VUS	Tier3	1/12 (8)
STK11	Chr19: 1220467	NM_000455.5 c.557_560delins p.Thr186Asnfs*4	Nonsense Exon: 4	Novel	Likely pathogenic	Tier2	1/12 (8)
STK11	Chr19: 1221314	NM_000455.5 c.842del p.Pro281Argfs*6	Frame_Shift_Del	rs121913321	Pathogenic	Tier2	2/12 (17)

**FIGURE 3:**
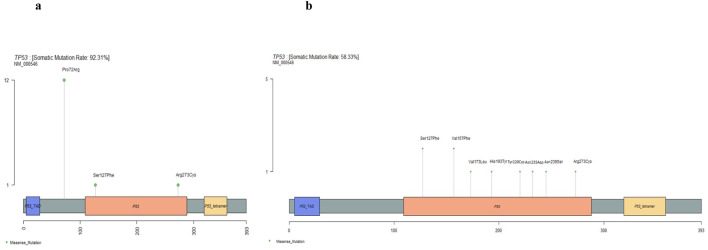
TP53 germline and somatic mutation profiles. Lolliplot showing the distribution of germline **(a)** and somatic **(b)** missense mutations identified in the TP53 gene in TNBC cohort.

The most frequently altered gene was TP53, with pathogenic or likely pathogenic missense variants observed in 58% of patients, predominantly affecting exons 5–8, which encode the DNA-binding domain (DBD). Notable recurrent TP53 variants within the DBD included p.Val173Leu, p.Val157Phe, p.Ser127Phe, p.Arg273Cys, p.Tyr220Cys, p.His193Tyr, p.Asn239Ser, as well as a novel splice region deletion (c.560-6_560-1del) identified in our cohort (Figure 3). Other frequently mutated tumor suppressors and oncogenes included APC, STK11, SMAD4, RET, CDKN2A, MET, and VHL, with mutation frequencies ranging from 8% to 33%, highlighting recurrent alterations in key cancer-related pathways (Figure 2b). This profile underscores the concerted disruption of central tumor suppressor and oncogenic pathways, including p53 signaling, Wnt/β-catenin, and cell cycle regulation, in this TNBC cohort.

Variant type analysis revealed that missense mutations were the most common somatic alteration, followed by frameshift insertions/deletions, nonsense mutations, in-frame indels, and splice site alterations (Figure 2c). Tier 1 (pathogenic or likely pathogenic) mutations were identified in key oncogenes and tumor suppressors, including TP53, STK11, RET, and VHL, highlighting potential driver events that may contribute to TNBC tumorigenesis and progression (Figure 2b).

Correlations between the mutational profile and treatment response were observed. Several genes harbored multiple independent somatic variants in patients with differing therapeutic outcomes. Variants in APC, SMAD4, RET, and ATM were detected in patients who achieved a partial response to treatment (pCR >50%) but did not reach a complete response. In contrast, multiple missense variants in TP53 and single missense variants in CDH1, FGFR2, and KIT were predominantly found in patients with poor treatment response (pCR <50%). Notably, an in-frame deletion in NOTCH1 (p.Val1578del) was exclusively detected in two patients who later developed disease recurrence, one at 1 year and the other at 5 years, indicating a possible link between this variant and long-term relapse risk. Furthermore, a missense mutation in AKT1 (p.Glu17Lys, exon 4) co-occurred with the TP53 pathogenic variant p.Val173Leu in a treatment-resistant tumor (pCR = 0), highlighting a potential cooperative effect involving AKT1–TP53 axis dysfunction in mediating complete resistance (Figure 2b). These findings indicate that the distribution and type of somatic variants may correlate with treatment response in TNBC, highlighting candidate genes that could influence therapeutic outcomes and potentially serve as predictive biomarkers for relapse and resistance.

### Protein-Protein Interaction (PPI) Network Analysis

To elucidate the functional relationships among altered genes in our TNBC cohort, we constructed separate PPI networks for germline and somatic mutations using the STRING database.

**FIGURE 4:**
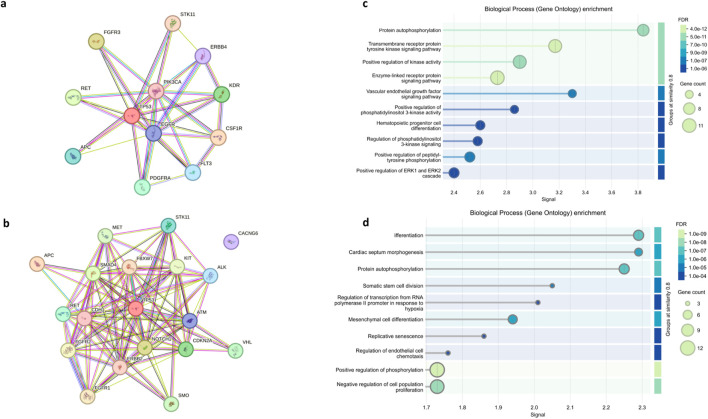
Protein–protein interaction (PPI) networks and GO pathway enrichment analysis of germline and somatic mutated genes in the TNBC cohort. **(a,b)** PPI networks illustrating functional interactions among germline and somatic variant–associated genes. **(c,d)** GO enrichment analysis highlighting significantly enriched biological processes, including receptor tyrosine kinase signaling, phosphorylation-related pathways, and cell differentiation. Node size and color reflect gene counts and enrichment significance (FDR), respectively.

The germline network, built from recurrently mutated genes (frequency >50%), comprised 12 nodes and 34 edges, significantly more than the 13 expected by chance (PPI enrichment p-value <0.001). This network was highly interconnected, with an average node degree of 5.67 and a clustering coefficient of 0.817, and featured TP53 as a central hub (Figure 4a).

The somatic network, incorporating all deleterious mutations, demonstrated even greater connectivity. Its 19 nodes and 102 edges (expected: 27; p < 0.001) formed a dense architecture, evidenced by an average node degree of 10.7 and a clustering coefficient of 0.799. TP53 again emerged as a central hub, underscoring its pivotal role in TNBC tumorigenesis across both genetic layers (Figure 4b).

Cluster analysis using the MCL identified a major functional module in both germline and somatic networks, significantly enriched for pathways including central carbon metabolism in cancer and transmembrane receptor protein tyrosine kinase activity, indicating coordinated involvement of these altered genes in metabolic regulation and oncogenic signalling pathways.

### Gene Ontology (GO) Enrichment Analysis

Gene Ontology (GO) enrichment analysis of germline-mutated genes revealed a highly significant overrepresentation of pathways associated with growth factor signalling and kinase-mediated regulatory mechanisms (Figure 4c). The top enriched Biological Process terms (False Discovery Rate, FDR <1.0 × 10^−9^) included transmembrane receptor protein tyrosine kinase signalling pathway, positive regulation of cell proliferation, and regulation of MAPK cascade. A considerable proportion of germline variants mapped to these signalling-related categories, suggesting an inherent predisposition to perturb key regulatory pathways governing cellular growth, survival, and proliferation. These findings indicate that inherited alterations may prime oncogenic signalling networks, thereby creating a favourable molecular context for TNBC initiation or early tumour development.

In contrast, GO enrichment analysis of somatically mutated genes revealed a strong overrepresentation of pathways involved in cell cycle control and maintenance of genomic integrity (Figure 4d). The most significantly enriched Biological Process terms (FDR <1.0 × 10^−9^) included mitotic cell cycle process, DNA repair, and response to DNA damage stimulus. Notably, a high number of somatically altered genes contributed to these key pathways (ranging from 9 to 12 genes per term), reflecting a concerted disruption of mechanisms essential for accurate cell division and genome stability. This pattern underscores that in TNBC, somatic mutations predominantly target processes driving uncontrolled proliferation and defective DNA damage responses, thereby promoting tumour progression.

## Discussion

In this study, we employed targeted NGS to characterize both germline and somatic mutations using a 50-gene cancer hotspot panel enriched for clinically relevant regions defined by the Catalogue of Somatic Mutations in Cancer (COSMIC). This approach enabled the precise detection of recurrent oncogenic alterations and facilitated a comprehensive molecular characterization of TNBC in a Tunisian cohort. Furthermore, it allowed us to investigate the potential associations between these genetic alterations and key clinicopathological features, including the response to neoadjuvant therapy.

### Germline Mutational Landscape

Genetic analysis of germline variants in breast cancer, including TNBC, has traditionally focused on pathogenic variants in *BRCA1/2*, as these genes are well-established drivers of hereditary breast cancer. However, other oncogenes and tumor-suppressor genes remain comparatively underexplored, despite growing evidence suggesting their important contribution to breast carcinogenesis [14]. In this study, we identified a spectrum of recurrent germline variants across several cancer-associated genes in patients with TNBC, including *TP53, CSF1R, FGFR3, RET, KDR, PDGFRA, APC, FLT3, EGFR, ERBB4, PIK3CA, MET,* and *ABL1.*

In the absence of a matched control group, distinguishing TNBC-specific germline variants from population-level polymorphisms remains challenging. To partially address this limitation, we compared variant frequencies observed in our cohort with allele frequencies reported in the Genome Aggregation Database (gnomAD). Several variants identified in our cohort are also present at high frequencies in the general population, including *FGFR3* (rs7688609, 100% vs. 99%), *PDGFRA* (rs1873778, 92% vs. 99%), *CSF1R* (rs2066933, 100% vs. 77%), and *FLT3* (rs2491231, 83% vs. 74%). Similarly, relatively high frequencies were observed for *RET* (rs1800861, 92% vs. 76%), *TP53* (rs1042522, 100% vs. 70%), *APC* (rs41115, 75% vs. 62%), and *EGFR* (rs1050171, 75% vs. 56%) compared with gnomAD. These observations suggest that many of these variants likely represent common germline polymorphisms rather than TNBC-specific susceptibility variants.

In contrast, other variants identified in our cohort appear at markedly lower frequencies in gnomAD, including *KDR* (rs7692791, 83% vs. 54%), *ERBB4* (rs839541, 58% vs. 26%), *PIK3CA* (rs2230461, 33% vs. 6%), *STK11* (rs2075606, 50% vs. 25%), *MET* (rs35775721, 42% vs. 5%), and *ABL1* (rs754813636, 25% vs. <0.001). Although these variants cannot be definitively associated with TNBC susceptibility without a matched control group, their relatively lower population frequencies may suggest a potential contribution to disease risk or tumor biology in this population.

Given the limited availability of genomic data for North African populations, we compared our findings with those recently reported by our group in a hepatocellular carcinoma (HCC) cohort analyzed using the same sequencing panel and analytical pipeline [15]. Several germline alterations were detected in both studies, although their frequencies differed between the two cancer types. These observations may indicate that these variants represent common polymorphisms within this population, or alternatively, that their combined presence reflects a genetic background that could influence cancer susceptibility.

Notably, several of these germline variants identified in both cancer types—including *FGFR3* (rs7688609, COSM4533173), *PDGFRA* (rs1873778, COSM7410554), *RET* (rs1800861, COSM4418405), *TP53* (rs1042522, COSM250061), *APC* (rs41115, COSM3760869), *EGFR* (rs1050171, COSM1451600), *KDR* (rs1870377, COSM149673), *ERBB4* (rs839541, COSM19690034), *PIK3CA* (rs2230461, COSM328028), *STK11* (rs2075606, COSM6666958), and *MET* (rs35775721, COSM1579024) — have already been reported as somatic mutations in the COSMIC database. Although several of these variants are classified as synonymous substitutions and are generally considered likely benign, they have nevertheless been reported to be implicated in key molecular pathways involved in the development of multiple cancer types. Examples include FGFR3 (rs7688609) in glioblastoma [16], RET (rs1800861) in thyroid carcinoma [17], KDR (rs7692791) in HCC [18], APC (rs41115) in adenomatous polyposis [19], and FLT3 (rs2491231) in TNBC [20].

### Key Germline Variants on Interest

The most frequently detected germline variant in our cohort was the well-characterized missense variant Pro72Arg (rs1042522, COSM250061) located in exon 4 of the tumor suppressor gene TP53. This variant, which results in a proline-to-arginine substitution at codon 72, is among the most widely distributed TP53 variants and has been reported across numerous cancer types, including breast, lung, colorectal, ovarian, and hepatocellular carcinoma in several ethnic [21]. Functionally, two isoforms exhibit distinct biological properties. The Arg72 (R72) variant has been shown to induce apoptosis, as well as influence cell migration, invasion, and metastatic potential more effectively than the Pro72 (P72) variant. Conversely, the P72 isoform is associated with enhanced DNA-repair capacity and increased cellular survival under genotoxic stress [22, 23]. In breast cancer, the presence of mutant p53 carrying the R72 variant has been significantly associated with poorer clinical outcomes [24]. Furthermore, recent case–control studies conducted in the Tunisian population have reported associations between this polymorphism and increased risk to chronic lymphocytic leukemia and cervical cancer [25, 26]. These findings indicate that, while not classified as pathogenic, Pro72Arg warrants further investigation to clarify its potential modulatory role in cancer susceptibility and tumor behaviour.

Additionally, we identified two recurrent 3′UTR variants in CSF1R gene (rs2066934 and rs2066933) in all samples of our cohort. Variants in the 3′UTR can alter post-transcriptional regulation by creating or disrupting microRNA recognition elements (MREs), thereby modulating mRNA stability and translation; In silico analyses have specifically predicted that rs2066934 may affect binding of numerous miRNAs and thus has the potential to substantially alter CSF1R expression [27]. Notably, growing evidence from high-throughput cancer sequencing studies indicates that these two CSF1R polymorphisms (rs2066934 and rs2066933) occur at relatively high frequencies, highlighting their emerging relevance in cancer genomics [28–30].

Taken together, the recurrent detection of these hotspot polymorphisms across different cancers highlights the need for well-designed case-control studies and underscores the importance of investigating potential gene-gene interactions to better clarify the contribution of these variants to cancer susceptibility.

### Pathway Enrichment and Functional Implications

Furthermore, PPI enrichment analysis revealed that the set of germline-altered genes identified in our study is significantly involved in major oncogenic signaling pathways. Notably, these genes clustered within the central carbon metabolism in cancer and PI3K/AKT signaling pathways, both crucial for metabolic reprogramming and survival in cancer cells [31]. Metabolic reprogramming, including enhanced glycolysis and glutaminolysis, is now considered a hallmark of cancer and supports anabolic processes and adaptation to stress [32]. In addition, enriched biological processes in our data set included protein autophosphorylation, transmembrane receptor protein tyrosine kinase signalling, and the VEGF signalling pathway, which regulate activation of receptor tyrosine kinases mechanisms frequently implicated in tumor progression and angiogenesis [33]. The involvement of these processes suggests that germline variants may modulate not only oncogenic signalling but also angiogenic responses and cellular proliferation. Collectively, these subtle germline alterations, despite being often labelled as benign, might perturb critical signalling networks and contribute to cancer susceptibility by priming cells for enhanced responsiveness to oncogenic stimuli.

### Somatic Mutational Landscape and Therapeutic Response

In addition to germline alterations, we investigated the spectrum of somatic mutations and their potential association with clinical features, particularly response to neoadjuvant therapy. As expected, TP53 emerged as the most frequently mutated gene, with nine deleterious alterations detected in 58% of tumor samples, consistent with its well-established role as the dominant driver of TNBC [34]. These missense mutations, all located within the DNA-binding domain (DBD), impair the oncosuppressive function of p53. These alterations were classified into two major categories with distinct functional and clinical implications. Mutations affecting residues directly involved in DNA interaction were classified as ‘contact mutants’. In contrast, substitutions such as V173L, R175H, and Y220C represent ‘structural mutants’, which destabilize the core DBD architecture, leading to its misfolding under physiological conditions [35, 36]. Critically, this structural misfolding is strongly associated with dominant-negative effects over p63 and p73 function and gain-of-function activities, which together confer enhanced resistance to chemotherapy-induced apoptosis compared to contact mutants [37, 38].

Our findings align with this functional dichotomy. Contact mutations such as p.Val157Phe, p.Arg273Cys, and p.Asn235Asp were detected in patients who achieved a pathologic complete response, while p.Asn239Ser was observed in a patient with a good therapeutic response. Moreover, multiple deleterious TP53 alterations, including p.His193Tyr, p.Ser127Phe, p.Val157Phe, and a novel splice-region deletion (c.560-6_560-1del), were only detected in patients with partial responses. Although these observations cannot establish a causal relationship, they may reflect cumulative structural destabilization and dominant-negative effects that impede chemotherapy efficacy.

In contrast to the favourable responses observed with contact mutations, structural TP53 mutants showed a markedly different pattern in our cohort. Notably, the p.Tyr220Cys (Y220C) substitution, one of the most destabilizing structural variants of the DBD, was identified in a patient who experienced tumor recurrence 1 year after treatment, consistent with the strong association of Y220C with impaired apoptotic signalling and poor clinical outcome [39]. Similarly, the p.Val173Leu (V173L, rs876660754) variant, another well-characterized structural mutant, was detected in a patient who exhibited complete resistance to neoadjuvant chemotherapy [36]. Moreover, previous studies have reported that the V173L variant co-occurred with an activating AKT1 (E17K, rs121434592) alteration, which may further promote pro-survival signalling and contribute to the observed chemo-resistance [40]. The AKT1 E17K mutation (rs121434592) has been widely reported to play an important role in tumor development and chemotherapy resistance across solid tumor types, including breast cancer [40, 41].

Furthermore, we identified the same NOTCH1 in-frame deletion (p.Val1578del) in both patients who relapsed. This variant has been reported to affect part of the PEST domain and is predicted to impair degradation of the NOTCH intracellular domain (NICD), leading to its prolonged nuclear persistence and sustained oncogenic signalling [42]. Notably, the patient who relapsed after 1 year also carried the TP53 Y220C structural mutant, suggesting a possible cooperative effect between dysfunctional p53 and persistent NOTCH1 activation that may contribute to early chemo-resistant relapse. In contrast, we hypothesize that the late recurrence at 5 years was driven solely by the NOTCH1 PEST-domain deletion, raising the possibility that NOTCH1 activation alone may sustain minimal residual disease and fuel long-term relapse. Nevertheless, they are consistent with previous reports indicating that TNBC harboring PEST-domain NOTCH1 mutations are sensitive to γ-secretase inhibitors [43]. Given the limited sample size, these observations should be interpreted with caution. Furthermore, comparison of the somatic mutations identified in this study with those reported in our previous work on HCC [15], reveals distinct mutational landscapes across the two tumor types, underscoring the context-specific nature of oncogenic alterations and highlighting the importance of cancer-tailored genomic screening strategies.

## Conclusion

In conclusion, our integrated analysis of germline and somatic alterations provides the first comprehensive overview of the complex molecular landscape of Tunisian TNBC patients using a targeted 50-oncogene panel. This study establishes a foundational genomic resource for North African TNBC patients and underscores the critical role of precision genomics in guiding personalized surveillance and therapeutic strategies in underserved populations.

### Limitations

This study has some limitations that should be acknowledged. First, our findings are derived from a single-institution cohort with a relatively small sample size, reflecting the low prevalence of TNBC (10%–15%) among breast cancer cases in our population. While this provides valuable preliminary insights, it may limit the generalizability of the results and introduce potential selection bias. Future studies involving larger, multi-center cohorts are required to validate and extend these findings.

Second, the use of a targeted gene panel, while clinically focused, and cost-effective, restricts the discovery of novel alterations outside the predefined regions. Notably, this panel does not cover key TNBC-associated genes such as *BRCA1/2*, which are critical for a complete characterization of TNBC mutational profiles in Tunisian cohort. Broader genomic approaches, such as whole-exome or whole-genome sequencing, would provide a more comprehensive understanding of the genetic determinants of TNBC and enable functional characterization of newly identified variants. Expanding sequencing efforts to larger cohorts and additional genomic regions and other breast cancer subtypes, will be essential for uncovering population-specific mutations and improving clinical management in Tunisian breast cancer patients.

## Summary Table

### What Is Known About This Subject

- TNBC is a highly aggressive breast cancer subtype with limited targeted therapeutic options.
- Germline and somatic variants play key roles in TNBC heterogeneity.
- Genomic data from North African populations remain limited.

### What This Paper Adds

- First exploratory profiling of germline and somatic variants in Tunisian TNBC patients.
- Identification of recurrent germline variants and heterogeneous somatic alterations.
- Descriptive insights into potential associations between mutation patterns and treatment response.

## Concluding Statement

This work represents an advance in biomedical science because it addresses a critical knowledge gap by providing preliminary genomic insights into TNBC in a North African population, establishing a foundation for targeted validation studies.

## Data Availability

The original contributions presented in the study are publicly available in the NCBI repository (https://www.ncbi.nlm.nih.gov/bioproject/PRJNA1454477), accession number PRJNA1454477.
